# m^6^A and cardiac posttranscriptional regulation: a novel player in heart development and disease

**DOI:** 10.1038/s12276-025-01528-8

**Published:** 2025-09-01

**Authors:** Donna Li, Deqiang Li, Jihyun Jang

**Affiliations:** 1 Center for Cardiovascular Research, Abigail Wexner Research Institute, Nationwide Children’s Hospital, Columbus, OH USA; 2 Department of Pediatrics, The Ohio State University College of Medicine, Columbus, OH USA; 3 Center for RNA Biology, The Ohio State University, Columbus, OH USA

**Keywords:** DNA methylation, Congenital heart defects

## Abstract

The growing body of research on cardiac epitranscriptomic factors has underscored their potential biological roles and impact on heart development and disease. While transcriptional and translational regulation of cardiac genes in the developing heart has been extensively studied, the potential roles of posttranscriptional regulation of cardiac mRNAs remain a significant gap in our understanding. In addition, discrepancies between transcriptomes and proteomes in both embryonic and adult hearts are well recognized, further suggesting an emerging regulatory role of the epitranscriptome in cardiac biology. Here we summarize the current understanding of m^6^A machinery and associated RNA-binding proteins in the heart and discuss their impact on heart development. By identifying existing knowledge gaps, we aim to provide insights that may inform future research directions.

## m6A modifications shape heart development and function

This Review explores how a specific chemical change in RNA, called m^6^A methylation, affects heart development and disease. m^6^A methylation is a process in which a small chemical group is added to RNA, influencing how genes are turned into proteins. The authors focus on how this process impacts the heart. The study reviews existing research on m^6^A methylation in both developing and adult hearts. It highlights that m^6^A plays a crucial role in heart function by regulating gene expression. Researchers discuss how m^6^A affects heart cells’ growth and response to stress, using various animal models to study these effects. Key findings show that disruptions in m^6^A can lead to heart developmental defect such as early-onset dilated cardiomyopathy. The research suggests that a deeper understanding of m^6^A could pave the way for novel therapeutic strategies for heart disease.

This summary was initially drafted using artificial intelligence, then revised and fact-checked by the author.

## Introduction

Posttranscriptional regulation is essential for gene expression, as it modulates RNA molecules after transcription but before translation^1^. This process determines which genes are expressed, the amount of protein produced and the timing of production. Various mechanisms contribute to posttranscriptional regulation, including RNA stability, splicing, transport, degradation and translation initiation. Disruptions in these regulatory pathways are linked to numerous human diseases^2^. Recent studies highlight how dysregulation of RNA-binding proteins and alterations in mRNA stability and processing contribute to disease pathogenesis, including cancer, neurodegenerative disorders and cardiovascular diseases^3,4^. A key aspect of posttranscriptional regulation involves biochemical modifications of RNA, which play a crucial role in gene expression. So far, over 170 RNA modifications have been identified in both coding and noncoding RNAs, including pseudouridine (Ψ), inosine (I), 5-methylcytosine (m^5^C) and various adenosine modifications^5^. Pseudouridine (Ψ) is formed through the isomerization of uridine, altering RNA structure^6^. mRNA pseudouridylation has been identified as a factor affecting RNA–RNA or RNA–protein interactions^7^. Inosine (I) is formed through adenosine-to-inosine RNA editing by hydrolytic deamination at the C6 position of adenine, catalyzed by the adenosine deaminase acting on RNA (ADAR) enzyme^8^. m^5^C, a cytosine modification, affects RNA stability and processing^9^. Adenosine modifications, such as *N*^1^-methyladenosine (m^1^A) and *N*^6^-methyladenosine (m^6^A), are distributed throughout mRNAs, including stop codons, untranslated regions (UTRs) and coding sequences^10^. Among them, m^6^A is the most prevalent and well-characterized modification in mammalian mRNAs and has been extensively studied due to its diverse functions^11,12^.

Similar to DNA and histone modifications, m^6^A methylation is dynamically regulated by methyltransferases, which add the methyl modification, and demethylases, which remove it^13^. After methylation, RNA-binding proteins recognize the m^6^A mark and mediate its effects^14^. m^6^A has been shown to affect gene expression and cellular behavior (for example, stem cell pluripotency^15^, cancer progression^16,17^ and metabolism^18,19^). Recent studies have highlighted the role of m^6^A modifiers in adult hearts under both normal and pathological conditions^20,21^. In the adult heart, m^6^A is critical for maintaining cardiac function, with increased levels associated with compensated hypertrophy and reduced levels linked to pathological remodeling and dysfunction^22^. Dysregulated m^6^A has been implicated in cardiovascular diseases such as heart failure, ischemic heart disease and arrhythmia by affecting key cellular processes, including cardiomyocyte differentiation, proliferation and apoptosis^23^. Although m^6^A modification is present in both adult and fetal tissues, the mechanisms driving adult-onset heart disease differ fundamentally from those underlying congenital heart disease, which originates from structural defects during fetal development. Genome-wide mapping of m^6^A across fetal organs, including the brain, liver, lung, kidney and heart, has attracted considerable interest for its insights into fetal development, lineage specification and tissue-specific functions^24^. Notably, 39.8% of m^6^A peaks in fetal tissues are found in the heart, where they are strongly enriched near splice sites, suggesting a regulatory role in RNA splicing^24^. Given m^6^A’s critical function in maintaining stemness and supporting embryogenesis^15,25^, it is likely to play a crucial role in heart formation. Notably, global knockout (KO) of m^6^A modifiers in mice results in early embryonic lethality due to reduced m^6^A levels^15,25,26^, hinting at potential defects in heart development. Moreover, recent studies reveal that m^6^A is indispensable not only during embryogenesis^27^ but also for postnatal cardiac maturation^28^. However, the precise functions of m^6^A methyltransferases in the developing myocardium remain poorly defined. As emerging evidence links aberrant m^6^A regulation to congenital heart defects^29,30^, further research into cardiac epitranscriptomics holds promise for uncovering the molecular underpinnings of these disorders. Drawing on PubMed-indexed studies published between 2001 and 2025, this Review examines the machinery governing m^6^A regulation, its influence on RNA metabolism in cardiac biology and its emerging role in heart development.

## The functional importance of m^6^A in RNA biology

**Fig. 1:**
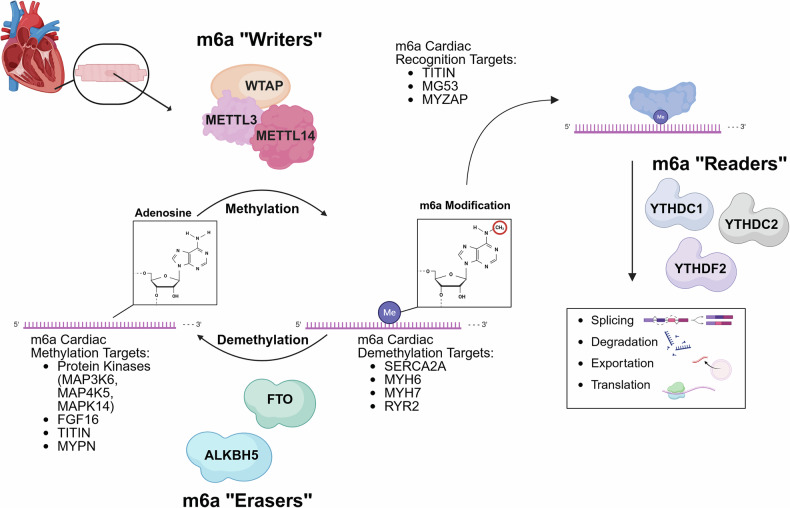
Illustration of in vitro and in vivo cardiac m^6^A expression conditions and their corresponding effects.

m^6^A modifications occur when a methyl group is added to the nitrogen at position 6 of the adenosine base in RNA. m^6^A occurs mostly in mRNAs, but recent increasing studies show that other RNA types such as ribosomal RNAs^31^, noncoding RNAs^32^ and transfer RNAs^33^ also undergo m^6^A modifications. m^6^A has emerged as a crucial posttranscriptional modification that influences a broad range of biological processes and is implicated in numerous cellular functions and diseases, including cancer, neurological disorders and cardiovascular diseases^23^. This modification is catalyzed by an intricate machinery known as the m^6^A methyltransferase complex, which plays an essential role in regulating various aspects of RNA metabolism, including RNA splicing, stability, translation and decay^12^. In both mammals and yeasts, m^6^A modification of RNA predominantly occurs within the consensus sequence RRACH (R = G or A; H = A, C or U) in gene-coding regions and 3′ UTRs, highlighting its essential role in RNA processing and translational regulation^34–37^. There are three key dynamic processes of m^6^A modification: writing (deposition), reading (recognition) and erasing (removal) of methyl from target RNAs, highlighting its reversible and dynamic nature (Fig. 1). First, the process is installed by the m^6^A methyltransferases ‘writers’ complex. Once m^6^A is deposited onto RNA, reading proteins, denominated ‘readers’, bind to the specific m^6^A-modified RNA to interpret and regulate its functions. Because this process is reversible, m^6^A can be removed by demethylases, termed ‘erasers’^38^. These modifications influence RNA processing and metabolism by using RNA-binding proteins to determine the fate of m^6^A-modified RNA^39^.

The m^6^A methyltransferase complex, responsible for most mRNA m^6^A modifications, consists of several writer subunits, including methyltransferase 3 (METTL3), methyltransferase 14 (METTL14) and Wilms Tumor 1 Associated Protein (WTAP)^12^. The methyltransferase complex is composed of catalytic proteins including METTL3 and METTL14, which are highly conserved in mammals and form a heterodimeric complex^40^ that recruits accessory partners^41^. The m^6^A modification is deposited co-transcriptionally by the METTL3–METTL14 complex. METTL3 is an *S*-adenosyl methionine (SAM)-binding protein, whereas METTL14 is critical to support the methyltransferase complex as an allosteric activator that binds to target RNAs^42–44^. Both METTL3 and METTL14 possess a methyltransferase domain, although METTL14 is catalytically inactive, as indicated by its lack of a SAM-binding site^42–44^. Rather, METTL14 primarily plays a role in methyltransferase complex stabilization and assists in RNA binding^44^, suggesting that METTL3 and METTL14 participate together in m^6^A RNA methylation^38^. WTAP has been identified as a regulatory subunit of the m^6^A methyltransferase complex. It binds to the METTL3–METTL14 complex and influences the methylation process, regulating m^6^A levels on RNA transcripts^38^. While recent reports highlight the important functions of METTL3 and METTL14 in adult hearts under both physiological and pathological conditions^20,21^, their potential roles during heart development are undetermined. WTAP has been identified as a regulator of pre-mRNA splicing through its interaction with the METTL3–METTL14 complex by accumulation of METTL3 and METTL14 in nuclear speckle^45^. *Wtap*-deficient mice exhibit embryonic lethality^46^, and *Wtap* knockdown in porcine models reduces global m^6^A levels and blastocyst formation^47^, underscoring the essential role of WTAP in early embryonic development.

The m^6^A readers recognize the selective binding site to the m^6^A-modified RNA and regulate their stability, splicing, translation and localization^12^ (Fig. 1). These readers are present in both the nucleus and cytoplasm, carrying out distinct functions depending on their cellular compartmentalization. Among m^6^A reader proteins, most possess a YT521-B homology (YTH) domain, which specifically recognizes the m^6^A modification, distinguishing it from unmodified adenosine of RNAs^48^. The function of each reader varies based on the positioning of m^6^A within the RNA. Nuclear m^6^A readers include the YTH *N*^6^-methyladenosine RNA-binding protein C1 (YTHDC1), which promotes exon inclusion in specific mRNAs by recruiting the pre-mRNA splicing factors^49^. Cytoplasmic readers include the YTHDF family (YTHDF1/2/3) and YTHDC2. Several studies propose distinct roles for these proteins. YTHDF1 enhances translation by interacting with translation initiation factors and ribosomes^50^. YTHDF2 was first identified through pull-down experiments using m^6^A-containing RNA probes^36^, and it has been known to facilitate mRNA decay by recruiting factors for the RNA decay machinery^50,51^. The biological function of YTHDF3 is more complex. YTHDF3 has been reported to enhance protein synthesis in collaboration with YTHDF1^52^, while it coordinates with YTHDF2 to regulate the decay of m^6^A-modified mRNA^52^. In addition, its overexpression promotes the expression of m^6^A-enriched transcripts that drive breast cancer brain metastasis^53^. The mechanism of YTHDC2 has been well studied in mammalian spermatogenesis. YTHDC2 is highly expressed in mouse testes, and it contains multiple RNA-binding domains and plays a dual role in RNA regulation. It enhances translation efficiency while simultaneously accelerating the decay of its target RNAs, contributing to efficient RNA turnover. This process allows the translation machinery to more effectively target remaining transcripts in differentiating cells^54^.

m^6^A erasers are enzymes responsible for removing the m^6^A modification from RNA, a process known as demethylation. These enzymes play a crucial role in the dynamic regulation of m^6^A levels, ensuring that RNA modifications are properly maintained and adjusted based on cellular needs. Several m^6^A erasers have been identified, including AlkB homolog 5 (ALKBH5) and fat mass and obesity-associated protein (FTO). ALKBH5 possesses an m^6^A binding pocket and plays a role in mRNA export, RNA metabolism and the organization of mRNA splicing factors within nuclear speckles^55^. *Alkbh5* KO in mice leads to increased m^6^A-induced apoptosis in spermatocytes^55^. In addition, inhibition of ALKBH5’s m^6^A demethylase activity results in DNA damage and apoptosis in response to reactive oxygen-induced stress^56^. FTO belongs to the AlkB family of nonheme Fe (II)/dioxygenases^57^ and mainly exerts a role in mRNA splicing in m^6^A-modified RNAs^58^, which has been associated with mouse embryonic stem cell development^59^, adipogenesis^58^ and enhanced melanoma tumorigenesis^60^ and cardiac heart repair^61^. FTO and ALKBH5 play crucial roles in the reversible modification of m^6^A, potentially adding an extra layer of biological regulation of various life processes, much like the modifications observed on DNA and histones.

## m^6^A methyltransferases in cardiac function and disease

The pioneering study on METTL3’s role in adult heart disease was conducted by Accornero’s group. Cardiomyocyte-specific deletion of *Mettl3* by *β-Mhc*-Cre in mice led to cardiac structural deterioration and dysfunction by around 8 months of age, without affecting heart development or early postnatal growth^20^. Meanwhile, enhancing m^6^A by overexpressing METTL3 in cardiomyocytes stimulates spontaneous hypertrophic growth, leading to compensated cardiac remodeling^20^. Under stress conditions, such as pressure overload induced by transverse aortic constriction or angiotensin II infusion, Cardiomyocyte-specific *Mettl3*-KO mice exhibited accelerated disease progression, highlighting METTL3’s essential role in cardiac adaptation to stress^20^. Mechanistically, the METTL3–m^6^A pathway predominantly methylates protein kinases, including MAP3K6, MAP4K5 and MAPK14^20^, which regulate kinase-driven signaling pathways responsible for hypertrophic growth^62^. The protein levels of these kinases were enhanced by *Mettl3* overexpression^20^. As *β-Mhc-*Cre is restricted to ventricular myocytes during development, further studies using chamber-specific or nonmyocyte Cre drivers may provide deeper insights into METTL3’s role in heart development. Another study highlighted METTL3’s function in postnatal cardiac growth. *Mettl3* expression increases at both mRNA and protein levels in neonatal myocardium between postnatal days 3 and 14, but this increase is abolished after neonatal heart injury, implicating METTL3 in postnatal heart growth^63^. *Mettl3* knockdown enhanced cardiomyocyte proliferation and accelerated heart regeneration after injury in both neonatal and adult mice, whereas its overexpression suppressed regeneration. Mechanistic analysis revealed that METTL3 enhances m^6^A modification of fibroblast growth factor 16 (FGF16) in a YTHDF2-dependent manner^63^. However, mutation of the m^6^A consensus sequence in *Fgf16* attenuated these effects, suggesting that m^6^A-modified FGF16 plays a key role in regulating postnatal cardiomyocyte proliferation^63^. In addition, recent findings suggested that METTL3 may play a role in regulating cardiac metabolic pathways in adult hearts^20^.

METTL14, another component of the methyltransferase complex, shares similar functions with METTL3 in the heart. Its perturbation reduces m^6^A levels and affects cardiac responses to ischemia–reperfusion (I/R) injury in neonatal cardiomyocytes^64^. *Mettl14* heterozygous mice showed notable cardiac infarct enlargement and aggravation of cardiac dysfunction after I/R^64^. By contrast, *Mettl14* overexpression reduced the cardiac infarct size and apoptosis, providing cardioprotective effects against I/R injury^64^. Mechanistically, *Mettl14* overexpression enhances m^6^A-modified Wnt family member 1 (WNT1) mRNA and protein expression, suggesting its role in WNT-dependent cardioprotection^64^. However, another study found that cardiac-specific *Mettl14* knockdown driven by cardiac Troponin T (*cTnnt2*) promoter alleviates cardiac dysfunction, reduces infarct size and mitigates the elevation of heart failure markers (for example, *Nppa*, *Nppb* and *β-Mhc*) after acute I/R injury^21^. In addition, exercise training was shown to downregulate METTL14 expression and m^6^A levels, leading to physiological cardiac hypertrophy^21^. This study also suggested that the METTL14 methyltransferase enzyme site is essential for the effect of METTL14 in both physiological and pathological conditions. The *Mettl14* heterozygous knockdown mice showed reduced levels of the protein in the heart tissue, resulting in enhanced cleaved caspase-3 protein expression, which suggests an acceleration in I/R-induced apoptosis^64^. The contrasting result of cardiac function alleviation from *Mettl14* inhibition could be attributed to the use of *cTnnt2-*Cre-driven downregulation as there are more than just cardiomyocytes in heart tissue. However, mutation of the catalytic site in METTL14 also disrupts the formation of the METTL3–METTL14 complex, highlighting the need for further investigation into its multifaceted role.

Our recent new study revealed the potential roles of methyltransferases in heart development including sarcomere maturation and organization^65^. Cardiomyocyte-specific conditional KO of either *Mettl3* or *Mettl14* using c*Tnnt2*-Cre in mice led to severe ventricular dilation and a rounded heart shape, deviating from the typical cone-like structure observed in wild-type hearts right after birth, hallmarks of early-onset dilated cardiomyopathy (DCM)^65^. These morphological abnormalities emerged as early as embryonic day (E) 14.5 and became more pronounced by E18.5, ultimately leading to early postnatal lethality^65^. In both *Mettl3*- and *Mettl14*-KO hearts, m^6^A-modified transcripts encoding key sarcomeric proteins were markedly reduced^65^. These m^6^A sites were often enriched in exonic regions of sarcomeric genes, suggesting a potential role in regulating transcript processing. Notably, splicing variants of *Titin*, a gene frequently mutated in DCM, were remarkably dysregulated. TITIN produces two main isoforms with distinct mechanical properties: N2BA, the more compliant fetal isoform, and N2B, the shorter and stiffer adult isoform. In mouse cardiomyocytes at E13.5, the N2B isoform is already present, albeit at lower levels than N2BA^66^. The *Mettl3* and *Mettl14* KO led to significantly reduced A-band lengths and absence of I-bands^65^, probably due to disrupted Titin expression and abnormal isoform switching. As TITIN links actin filaments to Z-discs and plays a critical role in maintaining sarcomere structure, its dysregulation probably contributes to impaired myocardial development. Although m^6^A-modified sarcomeric mRNAs may be differentially interpreted by specific m^6^A reader proteins, these findings suggest that METTL3 and METTL14 share an mRNA modification mechanism in the developing heart and play essential roles in regulating early sarcomerogenesis and myofibrillogenesis.

WTAP, later identified as another component of the m6A complex, functions as an adaptor protein for METTL3^67^. WTAP knockdown reduces m^6^A levels in human cells and is crucial for heart function and disease progression^67^. Cardiomyocyte-specific deletion of WTAP using *α-Mhc*-Cre resulted in severe DCM and postnatal lethality by day 8 due to heart failure^28^. WTAP deficiency reduced the protein levels of METTL3 and METTL14 and altered chromatin accessibility at myocyte enhancer factor 2a (*Mef2a*) and *Mef2c*, resulting in decreased gene expression of these key transcription factors and their targets in cardiac development^28^. Interestingly, WTAP overexpression in cardiomyocytes, in combination with the ER stress inhibitor 4-PBA, increased m^6^A levels but also induced apoptosis, suggesting a complex role in cardiac pathology^68^. WTAP and m^6^A modification levels are also elevated in hypoxia–reoxygenation-treated cultured rat cardiomyocytes (H9C2) and in I/R-injured rat hearts^68^, where they have been associated with accelerated myocardial injury progression^69,70^.

## m^6^A-recognizing RNA-binding proteins in the heart

The YTH domain containing proteins are the most predominant m^6^A reader proteins, and they can influence mRNA splicing, degradation and exportation, through mediating translation and decay^67^. A genetic mouse study by Gao et al. demonstrated that m^6^A readers, including *Ythdf1*, *Ythdf2* and *Ythdf3*, are dispensable for embryonic development and the normal adult heart. However, conditional KO of *Ythdc1* in cardiomyocytes using *α-Mhc*-Cre led to significant left ventricular chamber enlargement, resulting in major systolic dysfunction and a DCM phenotype by 8 weeks^30^. In addition, *Ythdc1* deletion caused reduced cardiac contractility, prolonged cardiomyocyte relaxation time and disordered sarcomere arrangement, all of which align with the characteristics of cardiac DCM^30^. Mechanistically, YTHDC1 recognizes m^6^A modifications in *Titin* mRNA, and its deletion affects *Titin* splicing, resulting in an increased N2BA/N2B ratio of *Titin*^30^. This is particularly interesting for two reasons. First, *Titin* mutation is the most prevalent genetic contributor to the pathogenesis of DCM^71^. Second, among two major *Titin* isoforms, the shorter and stiffer N2B isoform is dominant in the adult heart, whereas N2BA, the more compliant isoform, is predominantly expressed under disease conditions, showing decreased passive tension leading to less diastolic forces^72^. These findings suggest that m^6^A modification of TITIN may be a critical regulatory target of YTHDC1, and its disruption may contribute to alternative splicing changes that underlie the development of DCM. A previous study showed that YTHDC1 knockdown led to decreased proliferation of human induced pluripotent stem cell-derived cardiomyocyte, suggesting that YTHDC1 regulates cardiomyocyte proliferation^73^.

Accumulated studies have highlighted the regulatory changes in YTHDF2 associated with cardiomyopathy, suggesting its critical role in the heart^74–76^. The loss of YTHDF2 impacts cardiac remodeling, as cardiomyocyte-specific deletion of Ythdf2 using *α-Mhc*-Cre mice accelerates age-related cardiac dysfunction, accompanied by increased hypertrophic and fibrotic markers^77^. YTHDF2 binds to m^6^A-modified MYZAP, an intercalated disc myocardial zonula adherens protein, with its deficiency leading to upregulation of MYZAP in cardiomyocytes and promoting its degradation^78^. YTHDC2 has recently been linked to cardiac hypertrophy through its interaction with zinc finger protein 36 (ZFP36)^79^. As a key ferroptosis regulator, ZFP36 has been shown to alleviate ferroptosis and reduce hypertrophy. However, when its expression is reduced, cardiac hypertrophy is promoted through the YTHDC2-dependent ferroptosis pathway^79^. YTHDF2 may hinder the protective effect against myocardial infarction. A recent study demonstrated that overexpression of MG53 in infarction-injury-induced rats reduced the myocardial infarction area, while overexpression of *Ythdf2* reversed this effect, abrogating the protective role of MG53 in cardiomyocytes, which are primarily expressed upon myocardial infarction^80^. These studies highlight important functions of YTHDF2 upon cardiac injuries, although additional in vivo cardiac functional studies need to be performed to understand its functions and mechanisms under various physiological and pathological conditions.

## m^6^A demethylases in cardiac function and disease

Besides m^6^A writers, m^6^A levels are also modulated by its demethylase enzymes, m^6^A erasers. Most of the functions of demethylases have been studied in the adult heart following injury. By utilizing human, pig and mouse models, FTO has been identified to play an important functional role in cardiac homeostasis and myocardial repair^61^. In failing mammalian hearts and hypoxic cardiomyocytes, the expression of FTO is decreased, accompanied by an increase in m^6^A in RNA^61^. Notably, increasing FTO expression in failing mouse hearts alleviates the m^6^A increase and cardiac contractile function decline induced by ischemia by preventing their degradation^61^. Both knockdown and global KO of *Fto* led to proarrhythmic remodeling in mouse hearts^61,81^, suggesting that FTO is essential for maintaining normal cardiac function. In myocardial infarction, adeno-associated virus 9 (AAV9) and adenovirus-mediated overexpression of *Fto* decreased the myocardial infarction-induced increase of m^6^A and improved cardiac function^61^. FTO was also discovered to demethylate sarcoendoplasmic reticulum calcium transport ATPase 2a (SERCA2A), myosin heavy chain 6/7 (MYH6/7) and ryanodine receptor 2 (RYR2), which regulate cardiac contractile properties, leading to increased mRNA and protein expression of these genes^61^.

Another notable player among m^6^A erasers is ALKBH5, which plays a dual role in regulating cardiomyocyte behavior after myocardial injury^73^. Although *Alkbh5* global KO mice do not exhibit noticeable abnormalities in cardiac morphology and function, their hearts show impaired regenerative capacity and reduced function after injury, such as apex resection or myocardial infarction, compared with wild-type controls^73^. By contrast, forced expression of *Alkbh5* considerably improves cardiac outcomes after injury by reducing infarct size, restoring heart function and promoting cardiomyocyte proliferation in both adult and juvenile mice^73^. Mechanistically, ALKBH5-mediated m^6^A demethylation enhances YTHDF1 expression by stabilizing its mRNA, thereby promoting the translation of Yes-associated protein (YAP), a key factor in cardiomyocyte proliferation^73^. Interestingly, other studies have revealed a contrasting role for ALKBH5 in pathological cardiac remodeling. In response to hypertrophic stimuli such as transverse aortic constriction or phenylephrine injection, Alkbh5 expression is upregulated^62^. In this condition, ALKBH5 promotes cardiomyocyte hypertrophy rather than proliferation. Knockdown of Alkbh5 leads to reduced cell size, while its overexpression enhances hypertrophic markers through activation of the JAK2–STAT3 pathway in an m^6^A-dependent manner^62^. These findings suggest that ALKBH5 activates distinct molecular programs depending on the physiological or pathological context: driving proliferation after injury, but hypertrophy under stress. Together, these observations highlight the complex and context-dependent functions of m^6^A erasers in the heart. While ALKBH5 contributes to both regenerative and hypertrophic responses, the viability of KO mice for m^6^A erasers into adulthood suggests that these enzymes may be nonessential during embryonic and early postnatal development, yet remain crucial for adapting to cardiac stress and injury.

## RNA-binding proteins as potential m^6^A readers in heart development

General RNA-binding proteins, which not only recognize m^6^A, have been extensively studied in the cardiovascular field^82–84^. Specifically, several cardiac-enriched RNA-binding proteins, such as RBFOX2^85^, SRSF2^85^, RBM20^86^ and RBPMS5^87^, are essential for heart development, some of which might be accomplished through their capacity to recognize m^6^A-modified RNAs^88^. These RNA-binding proteins preferentially associate with mRNA alternative splicing, which determines the appropriate levels of key splicing isoforms that are critical for normal heart development.

The RBFOX protein family, which includes RBFOX1 (A2BP1), RBFOX2 (RBM9) and the neuron-specific RBFOX3 (NeuN), is extensively studied for their essential roles in regulating alternative splicing^89^. All RBFOX proteins possess a single, highly conserved RNA recognition motif that specifically binds to the sequences UGCAUG and GCAUG^83,90,91^. Among them, RBFOX1 and RBFOX2 are key regulators of RNA splicing and are downregulated in heart diseases^84^. Notably, de novo loss-of-function mutations in RBFOX2 are strongly associated with hypoplastic left heart syndrome (HLHS), a severe congenital defect affecting the left side of the heart^92^. Indeed, the RBFOX2 nonsense mutation identified in patients with HLHS produces a truncated protein, disrupting its subcellular distribution and impairing RNA metabolism^93^. RBFOX2 is abundantly expressed in embryonic hearts, where it is present in both the nucleus and cytosol^94^. However, its expression declines in postnatal hearts as RBFOX1 levels increase^94^, highlighting its critical role in heart development. A genetic KO study of cardiac *Rbfox2* by the Kuyumcu-Martinez group revealed notable defects in yolk sac vasculature and cardiac chamber formation^85^. Cardiac *Rbfox2* KO dysregulates alternative splicing and disrupts cell adhesion to the extracellular matrix^85^. This impairment further affects endocardial–mesenchymal transition and cell cycle progression, both of which are critical for heart development^85^. Notably, these defects closely resemble those phenotypes observed in patients with HLHS. Beyond its role in regulating alternative splicing, RBFOX2 is also known to bind to the 3′ UTR of pre-mRNAs near the polyadenylation site, contributing to alternative polyadenylation^95^. Recently, the He group reported a new functional role for RBFOX2, finding that RBFOX2 binds to chromatin-associated RNAs and preferentially recognizes m^6^A-modified RNAs^88^. This interaction contributes to the recruitment of the RNA methyltransferase complex and chromatin regulators, such as RBM15 and PRC2. This mechanism opens a new avenue for understanding how RBFOX2, as an m^6^A reader, regulates transcription.

Serine/arginine-rich splicing factor 2 (SRSF2) contains an RNA recognition motif for binding RNA and a C-terminal domain rich in serine and arginine residues (RS domain) for binding other proteins^96^. It plays important roles in the regulation of constitutive and alternative splicing^96^. Germline deletions of *Srsf2* resulted in early embryonic lethality^97^. Cardiac-specific *Srsf2*-KO mice, generated using *Myl*-Cre, exhibit failure in postnatal heart development. These mice develop early-onset cardiomyopathy within 5 weeks after birth, along with aberrant excitation–contraction coupling^98^. Interestingly, SRSF2 interacts with m^6^A-containing RNAs without directly recognizing the m^6^A base, suggesting that SRSF2 may be recruited by different m^6^A effectors^58^. For example, the loss of FTO promotes m^6^A deposition and enhances SRSF2 binding, leading to increased inclusion of target exons^58^.

## Conclusion

mRNA m^6^A modifications may have distinct functions at different stages of heart development, under physiological or pathological conditions, as transcriptomes vary considerably in each of these contexts. As mentioned earlier, several studies have focused on the role of m^6^A modifiers in the adult heart under various pathological conditions, which differ greatly from their potential functions during embryonic and early postnatal heart development. As summarized in Table 1, current literature distinguishes the phenotypes observed from gene manipulations in both adult and developing hearts. Notably, there is limited research on m^6^A in developing cardiomyocytes, representing a distinct area of study compared with adult cardiomyocytes. Exploring the molecular mechanisms of m^6^A regulation in the developing heart could offer valuable insights into its role in congenital heart disease, underscoring the need for further investigation in this area.

**Table 1:** Phenotypic outcomes following gene perturbations in developing and adult hearts.

	Category	Genes	Phenotypes	Perturbations	Citation
**Developing hearts**	Methyltransferase	*Mettl3*	1. Severe ventricular dilation, postnatal lethality 2. Cardiac left ventricular malformation	1. CM-specific KO (*cTnnt2*-Cre) 2. CM-specific KO (*α-Mhc*-Cre)	^27,65^
	Methyltransferase	*Mettl14*	Severe ventricular dilation, Postnatal lethality	CM-specific KO (*cTnnt2*-Cre)	^65^
	Methyltransferase	*Wtap*	Severe DCM, neonatal death	CM-specific KO (*α-Mhc*-Cre)	^28^
	RNA-binding protein	*Ythdc1*	Left ventricular chamber enlargement	CM-specific KO (*α-Mhc*-Cre)	^30^
	RNA-binding protein	*Rbfox2*	Yolk sac vasculature and cardiac chamber formation defects, embryonic lethality	Cardiac progenitor KO (*Nkx2.5*-Cre)	^85^
**Adult hearts**	Methyltransferase	*Mettl3*	Maladaptive CM remodeling during aging and after stress	CM-specific KO (*β-Mhc*-Cre)	^20^
	Methyltransferase	*Mettl14*	Alleviation of acute I/R injury in the myocardium	CM-specific knockdown by AAV9	^21^
	Reader	*Ythdc1*	DCM characteristics and premature death at 10 weeks	CM-specific KO (*α-Mhc*-Cre)	^30^
	Reader	*Ythdf2*	1. Cardiac dysfunction, hypertrophy and fibrosis 2. Decreases heart function, cardiac remodeling after stress	1. CM-specific KO (*αMhc*-MerCreMer) 2. CM-specific KO (*α-Mhc*-Cre)	^77,78^
	Demethyltransferase	*Alkbh5*	Inhibition of cardiomyocyte proliferation upon injury	Global KO	^73^
	Demethyltransferase	*Fto*	Increased number of arrhythmic events	siRNA knockdown	^61^

*CM* cardiomyocyte.

The effect of m^6^A deposition on mRNA transcripts varies due to mechanisms that are not yet fully understood. While some universal effects exist, m^6^A modifications are context dependent and occur around a conserved motif in the stop codons, the 3′ and 5′ UTRs, and the coding sequence, including both intronic and exonic regions^99^. The position of the modification on the mRNA may determine which step of transcript processing is most affected by m^6^A. Moreover, specific m^6^A sites in cellular mRNA could be visualized once the exact modification site is determined^100^. For example, identifying the cardiac-specific mRNA loci with differential modification in a cardiomyocyte context could provide valuable insights. This would enable the investigation of how m^6^A-modified mRNA localization and stoichiometry change during heart development and disease progression in vivo.

Cardiac m^6^A mRNA modification has been shown to greatly influence both normal and pathological cardiac phenotypes under a variety of conditions, including developmental, stress-induced and I/R injury contexts. Its disruption has been associated with embryonic and postnatal lethality, as well as with functional impairments such as physiological cardiac defects. Each component of the m^6^A machinery plays a distinct role in heart development: the methyltransferase complex regulates signaling pathways involved in hypertrophic and proliferative growth; reader proteins interpret the m^6^A mark to influence alternative splicing, translation, RNA stability and export; and demethylases maintain cardiac homeostasis by fine-tuning these processes. Gene manipulation studies, including KO, knockdown and overexpression models, have revealed a spectrum of cardiac phenotypes. In vivo, these range from cardiac structural deterioration, ventricular dilation and disorganized sarcomere architecture, while in vitro models have shown increased apoptosis and impaired cardiomyocyte proliferation.

**Fig. 2:**
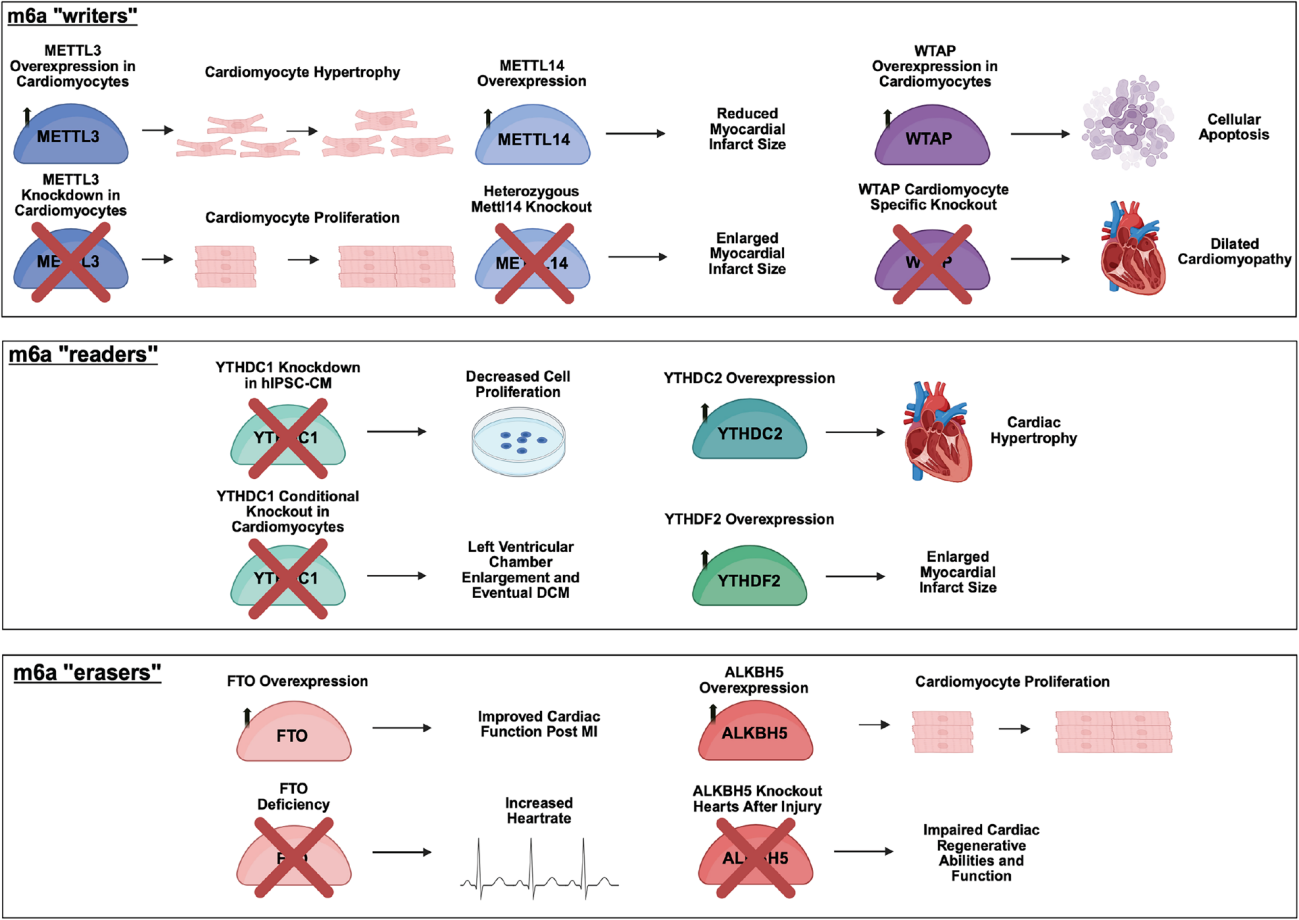
The cardiac m^6^A mechanism and previously studied targets.

Although numerous studies have demonstrated the substantial impact of m^6^A modifications and RNA-binding proteins on cardiac health and disease (Fig. 2 and Table 1), much of the focus has been on cardiomyocyte-related conditions, with limited understanding of their roles in noncardiomyocyte-related disorders. This gap in knowledge highlights the need for further research into the mechanisms underlying m^6^A and RNA-binding proteins in these areas. In the adult heart, beating cardiomyocytes are accompanied by a majority of nonmyocytes, including cardiac fibroblasts, endothelial cells and immune cells, which play crucial roles in providing structural support, maintaining homeostasis and facilitating injury repair^101^. Indeed, m^6^A modification has a crucial role in angiogenesis and vascular diseases, involving endothelial cells, their associated parietal cells, and immune cells^102^. These nonmyocytes are essential for overall cardiac function and are increasingly recognized as key contributors to heart health and disease^101^, suggesting the need for further studies to better understand these cell types. Nonmyocytes are also critical during embryonic heart development, where they help guide cardiomyocyte growth^13^. In general, nonmyocardial tissues and cells support cardiomyocyte proliferation, differentiation and myocardial patterning through the secretion of growth factors^13^. For example, cardiac endocardial cells, which are derived from early progenitor cells, provide essential paracrine signals and nutritional support for myocardial growth, as well as for the formation of coronary arteries and the maturation of cardiac valves^103–109^. The epicardium, the outermost mesothelial layer covering the heart, serves as a crucial hub for embryonic heart development^110,111^. During early heart development, the epicardium and its derivatives contribute to the formation of coronary vasculature, cardiac mesenchyme and support ventricular wall morphogenesis by providing paracrine signals^110–114^. Given the growing recognition of the importance of noncardiomyocyte populations, we suggest that future research should focus on these underexplored cell types to better understand their involvement in heart development and disease. Investigating the function of m^6^A and RNA-binding proteins in noncardiomyocytes could unveil new posttranscriptional mechanisms for a broader range of heart-related conditions, including those with congenital origins or noncardiomyocyte involvement.
